# 2-[(*E*)-4-Quinolylmethyl­ideneamino]­phenol

**DOI:** 10.1107/S1600536808033928

**Published:** 2008-10-22

**Authors:** Sema Öztürk Yıldırım, Mehmet Akkurt, Mustafa Kemal Gümüş, Mevlüde Canlıca, Şeniz Kaban, Vickie McKee

**Affiliations:** a Department of Physics, Faculty of Arts and Sciences, Erciyes University, 38039 Kayseri, Turkey; b Chemistry Department, Yıldız Technical University, Davutpasa Campus, Esenler 34220, Istanbul, Turkey; c Chemistry Department, Loughborough University, Loughborough, Leicestershire LE11 3TU, England

## Abstract

In the title compound, C_16_H_12_N_2_O, the dihedral angle between the two aromatic ring systems is 68.54 (5)°. The mol­ecular packing is stabilized by intra- and inter­molecular O—H⋯N and intra­molecular C—H⋯N hydrogen-bond inter­actions.

## Related literature

For bond-length data, see: Allen *et al.* (1987 ▶). For general background, see: Gao *et al.* (2005 ▶); Hagen *et al.* (1983 ▶); Lozytska *et al.* (2004 ▶); Sessler *et al.* (2004 ▶); Kuz’min *et al.* (2000 ▶). For related structures, see: Räisänen, Elo *et al.* (2007 ▶); Räisänen Leskelä & Repo (2007 ▶). For experimental procedures, see: Gümüş (2002 ▶).
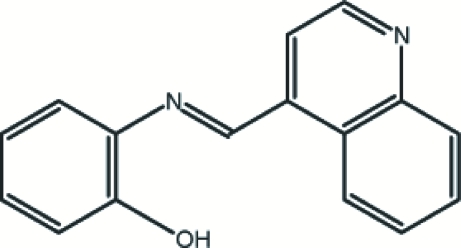


## Experimental

### 

#### Crystal data

C_16_H_12_N_2_O
*M* *_r_* = 248.28
Monoclinic, 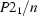

*a* = 6.7174 (6) Å
*b* = 23.931 (2) Å
*c* = 7.7495 (6) Å
β = 105.521 (1)°
*V* = 1200.33 (17) Å^3^
*Z* = 4
Mo *K*α radiation
μ = 0.09 mm^−1^
*T* = 150 (2) K
0.25 × 0.20 × 0.10 mm

#### Data collection

Bruker APEXII CCD diffractometer
Absorption correction: multi-scan (*SADABS*; Sheldrick, 2003 ▶) *T*_min_ = 0.978, *T*_max_ = 0.991
12294 measured reflections
2991 independent reflections
2338 reflections with *I* > 2σ(*I*)
*R*_int_ = 0.037

#### Refinement

*R*[*F* ^2^ > 2σ(*F* ^2^)] = 0.042
*wR*(*F* ^2^) = 0.105
*S* = 1.05
2991 reflections
220 parameters
H atoms treated by a mixture of independent and constrained refinement
Δρ_max_ = 0.25 e Å^−3^
Δρ_min_ = −0.24 e Å^−3^

### 

Data collection: *APEX2* (Bruker, 2005 ▶); cell refinement: *SAINT* (Bruker, 2005 ▶); data reduction: *SAINT*; program(s) used to solve structure: *SIR97* (Altomare *et al.*, 1999 ▶); program(s) used to refine structure: *SHELXL97* (Sheldrick, 2008 ▶); molecular graphics: *ORTEP-3 for Windows* (Farrugia, 1997 ▶); software used to prepare material for publication: *WinGX* (Farrugia, 1999 ▶).

## Supplementary Material

Crystal structure: contains datablocks global, I. DOI: 10.1107/S1600536808033928/hb2807sup1.cif

Structure factors: contains datablocks I. DOI: 10.1107/S1600536808033928/hb2807Isup2.hkl

Additional supplementary materials:  crystallographic information; 3D view; checkCIF report

## Figures and Tables

**Table 1:** Hydrogen-bond geometry (Å, °)

*D*—H⋯*A*	*D*—H	H⋯*A*	*D*⋯*A*	*D*—H⋯*A*
O1—H1⋯N1	0.95 (2)	2.34 (2)	2.7766 (16)	107.5 (15)
O1—H1⋯N2^i^	0.95 (2)	1.91 (2)	2.8085 (16)	156.4 (19)
C13—H13⋯N1	0.975 (15)	2.356 (14)	2.9776 (17)	121.0 (11)

Symmetry code: (i) 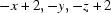.

**Fig. 1.**
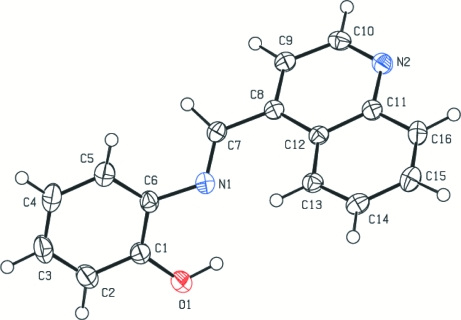
View of (I): displacement ellipsoids for non-H atoms are drawn at the 50% probability level.

**Fig. 2.**
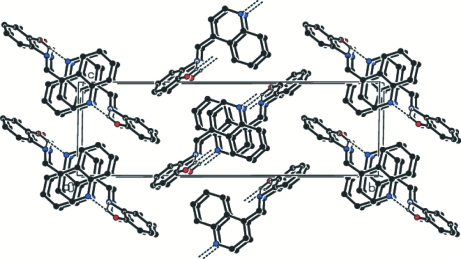
The hydrogen bonding interactions of (I) viewed down the a axis. For clarity, H atoms not involved in hydrogen bonding have been omitted.

## supplementary crystallographic information

### Comment

2- and 4-(*N*-arylimino)quinolines have been investigated to their clinical efficacy for use as an analgetic drug (Hagen *et al.*, 1983). A number of azomethines and their complexes possess high biological activity, including antineoplastic (Kuz'min *et al.*, 2000; Gao *et al.*, 2005) and antiviral (Lozytska *et al.*, 2004). It has been recently shown that the macrocyclic Schiff bases can selectively bind different anions acting as artificial anionic receptors (Sessler *et al.*, 2004).

In the title compound, (I), (Fig. 1), all bond lengths and angles are within normal ranges (Räisänen, Elo *et al.*, 2007; Räisänen Leskelä, & Repo 2007; Allen *et al.*, 1987). The molecule has a non-planar conformation. The dihedral angle between its two aromatic ring systems (C1–C6) and (N2/C8—C16) is 68.54 (5)°. The C6—N1—C7—C8 torsion angle is -179.98 (13)° and the N1—C6—C1—O1 angle deviates from zero only by -7.27 (18)°.

The molecular packing of the compound is stabilized by O—H···N and C—H···N hydrogen bonds (Table 1, Fig. 2).

### Experimental

Quinoline-4-carboxaldehyde (1.0 mmol) was dissolved in hot absolute ethanol (5 ml) and an equimolar amount of 2-aminophenol dissolved in hot absolute ethanol (10 ml) was added. The mixture was refluxed at reaction temperature for 2 h. The progress of the reaction was monitored by TLC using n-hexane/ethylacetate (1:1 *v*/*v*) as eluent. Upon completion of the reaction, the crude product which precipitated on cooling was collected by filtration. Further purification was accomplished by recrystallization from ethanol to yield yellow blocks of (I). [yield 80%, m.p. 475–476 K]. UV (CHCl_3_): λ_max_248, 343, 377 nm. IR (KBr): 3029, 2851, 1625, 1574 and1472, 1293–1165, 830 and 757 cm^-1. 1^H NMR (CDCl_3_, 200 MHz): δ(p.p.m.) 6.95–9.08 (m, ArH and CH, 11H), 9.41(s, OH, 1H). MS: m/*z* 249 (*M*+1), 248 (*M*+), 247 (*M*-1), 128 (*M*-120), 120 (*M*-128). Analysis calculated for C_16_H_12_N_2_O: C 77.40, H 4.87, N 11.28%; found: C 77.62, H 4.96, N 11.12% (Gümüş, 2002).

### Refinement

The H atoms were found from a difference map and refined freely.

### Crystal data

**Table:**

C_16_H_12_N_2_O	*F*(000) = 520
*M**_r_* = 248.28	*D*_x_ = 1.374 Mg m^−^^3^
Monoclinic, *P*2_1_/*n*	Mo *K*α radiation, λ = 0.71073 Å
Hall symbol: -P 2yn	Cell parameters from 2762 reflections
*a* = 6.7174 (6) Å	θ = 2.9–26.4°
*b* = 23.931 (2) Å	µ = 0.09 mm^−^^1^
*c* = 7.7495 (6) Å	*T* = 150 K
β = 105.521 (1)°	Block, yellow
*V* = 1200.33 (17) Å^3^	0.25 × 0.20 × 0.10 mm
*Z* = 4	

### Data collection

**Table:**

Bruker APEXII CCD diffractometer	2991 independent reflections
Radiation source: sealed tube	2338 reflections with *I* > 2σ(*I*)
graphite	*R*_int_ = 0.037
φ and ω scans	θ_max_ = 28.4°, θ_min_ = 1.7°
Absorption correction: multi-scan (SADABS; Sheldrick, 2003)	*h* = −8→8
*T*_min_ = 0.978, *T*_max_ = 0.991	*k* = −31→31
12294 measured reflections	*l* = −10→10

### Refinement

**Table:**

Refinement on *F*^2^	Primary atom site location: structure-invariant direct methods
Least-squares matrix: full	Secondary atom site location: difference Fourier map
*R*[*F*^2^ > 2σ(*F*^2^)] = 0.042	Hydrogen site location: difmap and geom
*wR*(*F*^2^) = 0.105	H atoms treated by a mixture of independent and constrained refinement
*S* = 1.05	*w* = 1/[σ^2^(*F*_o_^2^) + (0.0424*P*)^2^ + 0.3303*P*] where *P* = (*F*_o_^2^ + 2*F*_c_^2^)/3
2991 reflections	(Δ/σ)_max_ < 0.001
220 parameters	Δρ_max_ = 0.25 e Å^−^^3^
0 restraints	Δρ_min_ = −0.24 e Å^−^^3^

### Special details

**Table:**

Geometry. Bond distances, angles *etc*. have been calculated using the rounded fractional coordinates. All su's are estimated from the variances of the (full) variance-covariance matrix. The cell e.s.d.'s are taken into account in the estimation of distances, angles and torsion angles
Refinement. Refinement on *F*^2^ for ALL reflections except those flagged by the user for potential systematic errors. Weighted *R*-factors *wR* and all goodnesses of fit *S* are based on *F*^2^, conventional *R*-factors *R* are based on *F*, with *F* set to zero for negative *F*^2^. The observed criterion of *F*^2^ > σ(*F*^2^) is used only for calculating -*R*-factor-obs *etc*. and is not relevant to the choice of reflections for refinement. *R*-factors based on *F*^2^ are statistically about twice as large as those based on *F*, and *R*-factors based on ALL data will be even larger.

### Fractional atomic coordinates and isotropic or equivalent isotropic displacement parameters (Å^2^)

**Table:**

	*x*	*y*	*z*	*U*_iso_*/*U*_eq_	
O1	1.06305 (16)	0.13183 (4)	0.54890 (14)	0.0340 (3)	
N1	0.76446 (16)	0.10349 (4)	0.72156 (14)	0.0243 (3)	
N2	0.80975 (16)	−0.03956 (5)	1.22446 (15)	0.0258 (3)	
C1	0.8930 (2)	0.16414 (5)	0.52971 (17)	0.0264 (4)	
C2	0.8697 (3)	0.20982 (6)	0.41486 (19)	0.0340 (4)	
C3	0.6997 (3)	0.24448 (6)	0.3910 (2)	0.0372 (5)	
C4	0.5529 (3)	0.23493 (6)	0.4839 (2)	0.0370 (5)	
C5	0.5745 (2)	0.18955 (6)	0.59862 (19)	0.0313 (4)	
C6	0.7433 (2)	0.15362 (5)	0.62205 (17)	0.0242 (4)	
C7	0.72483 (19)	0.10243 (5)	0.87303 (17)	0.0235 (3)	
C8	0.74355 (18)	0.05168 (5)	0.98390 (16)	0.0215 (3)	
C9	0.78135 (19)	0.05904 (6)	1.16557 (17)	0.0236 (3)	
C10	0.81518 (19)	0.01260 (6)	1.28017 (17)	0.0260 (4)	
C11	0.76319 (18)	−0.04841 (5)	1.04285 (17)	0.0233 (3)	
C12	0.72937 (18)	−0.00427 (5)	0.91566 (16)	0.0212 (3)	
C13	0.67854 (19)	−0.01837 (6)	0.73121 (17)	0.0240 (4)	
C14	0.6654 (2)	−0.07303 (6)	0.67751 (18)	0.0278 (4)	
C15	0.7014 (2)	−0.11657 (6)	0.8042 (2)	0.0308 (4)	
C16	0.7487 (2)	−0.10454 (5)	0.98290 (19)	0.0282 (4)	
H1	1.068 (3)	0.1013 (9)	0.628 (3)	0.072 (6)*	
H2	0.976 (3)	0.2161 (7)	0.350 (2)	0.044 (5)*	
H3	0.686 (3)	0.2753 (7)	0.307 (2)	0.046 (5)*	
H4	0.433 (3)	0.2587 (7)	0.469 (2)	0.042 (5)*	
H5	0.468 (2)	0.1808 (6)	0.657 (2)	0.033 (4)*	
H7	0.689 (2)	0.1376 (6)	0.929 (2)	0.031 (4)*	
H9	0.788 (2)	0.0966 (6)	1.214 (2)	0.031 (4)*	
H10	0.844 (2)	0.0186 (6)	1.409 (2)	0.024 (4)*	
H13	0.653 (2)	0.0114 (6)	0.642 (2)	0.025 (4)*	
H14	0.632 (2)	−0.0822 (6)	0.550 (2)	0.032 (4)*	
H15	0.693 (2)	−0.1566 (7)	0.765 (2)	0.035 (4)*	
H16	0.772 (2)	−0.1340 (7)	1.074 (2)	0.033 (4)*	

### Atomic displacement parameters (Å^2^)

**Table:**

	*U*^11^	*U*^22^	*U*^33^	*U*^12^	*U*^13^	*U*^23^
O1	0.0385 (6)	0.0322 (5)	0.0359 (6)	0.0058 (4)	0.0181 (4)	0.0097 (4)
N1	0.0246 (5)	0.0223 (5)	0.0254 (6)	0.0006 (4)	0.0055 (4)	0.0023 (4)
N2	0.0220 (5)	0.0295 (6)	0.0259 (6)	0.0025 (4)	0.0062 (4)	0.0040 (5)
C1	0.0343 (7)	0.0221 (6)	0.0214 (6)	−0.0012 (5)	0.0049 (5)	−0.0020 (5)
C2	0.0475 (9)	0.0274 (7)	0.0272 (7)	−0.0040 (6)	0.0100 (6)	0.0022 (6)
C3	0.0549 (10)	0.0214 (7)	0.0308 (8)	−0.0020 (6)	0.0037 (7)	0.0037 (6)
C4	0.0436 (9)	0.0238 (7)	0.0383 (8)	0.0074 (6)	0.0018 (7)	0.0010 (6)
C5	0.0335 (7)	0.0257 (7)	0.0328 (7)	0.0021 (6)	0.0057 (6)	0.0002 (6)
C6	0.0303 (7)	0.0185 (6)	0.0217 (6)	−0.0016 (5)	0.0036 (5)	−0.0009 (5)
C7	0.0226 (6)	0.0221 (6)	0.0249 (6)	0.0003 (5)	0.0050 (5)	−0.0017 (5)
C8	0.0168 (6)	0.0238 (6)	0.0238 (6)	−0.0003 (5)	0.0052 (4)	0.0004 (5)
C9	0.0211 (6)	0.0251 (6)	0.0248 (6)	−0.0007 (5)	0.0064 (5)	−0.0023 (5)
C10	0.0227 (6)	0.0345 (7)	0.0210 (6)	0.0009 (5)	0.0060 (5)	0.0005 (5)
C11	0.0173 (6)	0.0259 (6)	0.0269 (6)	0.0004 (5)	0.0062 (5)	0.0017 (5)
C12	0.0166 (6)	0.0234 (6)	0.0241 (6)	0.0000 (4)	0.0064 (4)	−0.0004 (5)
C13	0.0212 (6)	0.0266 (7)	0.0247 (6)	0.0006 (5)	0.0070 (5)	0.0001 (5)
C14	0.0249 (6)	0.0297 (7)	0.0285 (7)	−0.0002 (5)	0.0068 (5)	−0.0056 (5)
C15	0.0282 (7)	0.0240 (7)	0.0388 (8)	0.0006 (5)	0.0064 (6)	−0.0036 (6)
C16	0.0257 (7)	0.0224 (6)	0.0358 (7)	0.0012 (5)	0.0071 (5)	0.0036 (6)

### Geometric parameters (Å, °)

**Table:**

O1—C1	1.3542 (17)	C11—C12	1.4210 (17)
O1—H1	0.95 (2)	C12—C13	1.4186 (18)
N1—C6	1.4126 (16)	C13—C14	1.368 (2)
N1—C7	1.2715 (17)	C14—C15	1.408 (2)
N2—C11	1.3740 (17)	C15—C16	1.366 (2)
N2—C10	1.3181 (19)	C2—H2	0.989 (19)
C1—C2	1.3918 (19)	C3—H3	0.972 (16)
C1—C6	1.4042 (19)	C4—H4	0.967 (19)
C2—C3	1.383 (3)	C5—H5	0.966 (14)
C3—C4	1.387 (3)	C7—H7	1.005 (15)
C4—C5	1.386 (2)	C9—H9	0.971 (15)
C5—C6	1.3957 (19)	C10—H10	0.975 (15)
C7—C8	1.4735 (17)	C13—H13	0.975 (15)
C8—C9	1.3734 (18)	C14—H14	0.978 (15)
C8—C12	1.4334 (17)	C15—H15	1.002 (17)
C9—C10	1.403 (2)	C16—H16	0.980 (16)
C11—C16	1.4161 (17)		
			
C1—O1—H1	113.2 (13)	C13—C14—C15	120.70 (13)
C6—N1—C7	120.37 (11)	C14—C15—C16	120.07 (13)
C10—N2—C11	117.40 (11)	C11—C16—C15	120.62 (12)
O1—C1—C2	117.77 (13)	C1—C2—H2	118.1 (10)
C2—C1—C6	119.56 (13)	C3—C2—H2	121.6 (10)
O1—C1—C6	122.67 (11)	C2—C3—H3	118.1 (12)
C1—C2—C3	120.27 (16)	C4—C3—H3	121.3 (12)
C2—C3—C4	120.54 (14)	C3—C4—H4	121.7 (10)
C3—C4—C5	119.68 (16)	C5—C4—H4	118.6 (10)
C4—C5—C6	120.53 (14)	C4—C5—H5	120.4 (9)
N1—C6—C1	116.70 (11)	C6—C5—H5	118.9 (9)
C1—C6—C5	119.40 (12)	N1—C7—H7	121.0 (8)
N1—C6—C5	123.63 (12)	C8—C7—H7	115.6 (8)
N1—C7—C8	123.26 (11)	C8—C9—H9	119.5 (9)
C7—C8—C12	124.61 (11)	C10—C9—H9	120.4 (9)
C9—C8—C12	118.28 (11)	N2—C10—H10	117.1 (9)
C7—C8—C9	117.10 (11)	C9—C10—H10	119.1 (9)
C8—C9—C10	120.11 (13)	C12—C13—H13	119.3 (9)
N2—C10—C9	123.87 (12)	C14—C13—H13	119.9 (9)
N2—C11—C16	117.33 (11)	C13—C14—H14	120.0 (9)
C12—C11—C16	119.56 (12)	C15—C14—H14	119.3 (9)
N2—C11—C12	123.11 (11)	C14—C15—H15	120.7 (9)
C8—C12—C13	124.63 (11)	C16—C15—H15	119.2 (9)
C11—C12—C13	118.22 (11)	C11—C16—H16	117.5 (9)
C8—C12—C11	117.13 (11)	C15—C16—H16	121.8 (9)
C12—C13—C14	120.82 (12)		
			
C7—N1—C6—C1	141.39 (13)	C7—C8—C9—C10	−175.57 (12)
C7—N1—C6—C5	−44.64 (19)	C12—C8—C9—C10	3.15 (19)
C6—N1—C7—C8	−179.98 (13)	C7—C8—C12—C11	176.39 (12)
C11—N2—C10—C9	−1.61 (19)	C7—C8—C12—C13	−5.5 (2)
C10—N2—C11—C12	2.52 (19)	C9—C8—C12—C11	−2.23 (18)
C10—N2—C11—C16	−177.36 (12)	C9—C8—C12—C13	175.91 (13)
O1—C1—C2—C3	−179.58 (13)	C8—C9—C10—N2	−1.3 (2)
C6—C1—C2—C3	−0.4 (2)	N2—C11—C12—C8	−0.62 (19)
O1—C1—C6—N1	−7.27 (18)	N2—C11—C12—C13	−178.87 (12)
O1—C1—C6—C5	178.49 (12)	C16—C11—C12—C8	179.26 (12)
C2—C1—C6—N1	173.54 (12)	C16—C11—C12—C13	1.00 (18)
C2—C1—C6—C5	−0.70 (19)	N2—C11—C16—C15	179.50 (13)
C1—C2—C3—C4	1.4 (2)	C12—C11—C16—C15	−0.4 (2)
C2—C3—C4—C5	−1.4 (2)	C8—C12—C13—C14	−179.03 (13)
C3—C4—C5—C6	0.4 (2)	C11—C12—C13—C14	−0.92 (19)
C4—C5—C6—N1	−173.12 (13)	C12—C13—C14—C15	0.2 (2)
C4—C5—C6—C1	0.7 (2)	C13—C14—C15—C16	0.4 (2)
N1—C7—C8—C9	153.65 (13)	C14—C15—C16—C11	−0.3 (2)
N1—C7—C8—C12	−25.0 (2)		

### Hydrogen-bond geometry (Å, °)

**Table:**

*D*—H···*A*	*D*—H	H···*A*	*D*···*A*	*D*—H···*A*
O1—H1···N1	0.95 (2)	2.34 (2)	2.7766 (16)	107.5 (15)
O1—H1···N2^i^	0.95 (2)	1.91 (2)	2.8085 (16)	156.4 (19)
C13—H13···N1	0.975 (15)	2.356 (14)	2.9776 (17)	121.0 (11)

Symmetry codes: (i) −*x*+2, −*y*, −*z*+2.

## References

[bb1] Allen, F. H., Kennard, O., Watson, D. G., Brammer, L., Orpen, A. G. & Taylor, R. (1987). *J. Chem. Soc. Perkin Trans. 2*, pp. S1–19.

[bb2] Altomare, A., Burla, M. C., Camalli, M., Cascarano, G. L., Giacovazzo, C., Guagliardi, A., Moliterni, A. G. G., Polidori, G. & Spagna, R. (1999). *J. Appl. Cryst.* **32**, 115–119.

[bb3] Bruker (2005). *APEX2* and *SAINT* Bruker AXS Inc., Madison, Wisconsin, USA.

[bb4] Farrugia, L. J. (1997). *J. Appl. Cryst.* **30**, 565.

[bb5] Farrugia, L. J. (1999). *J. Appl. Cryst.* **32**, 837–838.

[bb6] Gao, J., Ross Woolley, F. & Zingaro, R. A. (2005). *J. Med. Chem.* **48**, 7192–7197. 10.1021/jm050497t 16279777

[bb7] Gümüş, M. K. (2002). MSc thesis, Yıldız Technical University, Istanbul, Turkey.

[bb8] Hagen, V., Dove, B., Morgenstern, E., Labes, D., Göres, E., Tomaschewski, G., Geisler, G. & Franke, C. (1983). *Pharmazie*, **38**, 437–439. 6634910

[bb9] Kuz’min, V. E., Lozitsky, V. P., Kamalov, G. L., Lozitskaya, R. N., Zheltvay, A. I., Fedtchouk, A. S. & Kryzhanovsky, D. N. (2000). *Acta Biochim. Pol.* **47**, 867–875. 11310986

[bb10] Lozytska, R., Kryzhanovsky, D., Mazepa, A., Gorodniuk, V., Kuz’min, V., Lozitsky, V., Fedchuck, A., Rybalko, S., Diadium, S. & Vanden Eynde, J. J. (2004). *Arkivoc*, **xix**, 118R.

[bb11] Räisänen, M. T., Elo, P., Kettunen, M., Klinga, M., Leskelä, M. & Repo, T. (2007). *Synth. Commun.* **37**, 1765–1777.

[bb12] Räisänen, M. T., Leskelä, M. & Repo, T. (2007). *Acta Cryst.* E**63**, o1816–o1817.

[bb13] Sessler, J. L., Katayev, E., Pantos, G. D. & Ustynyuk, Yu. A. (2004). *J. Chem. Soc. Chem. Commun.* pp. 1276–1277. 10.1039/b403665d 15154034

[bb14] Sheldrick, G. M. (2003). *SADABS* Bruker AXS Inc., Madison, Wisconsin, USA.

[bb15] Sheldrick, G. M. (2008). *Acta Cryst.* A**64**, 112–122. 10.1107/S0108767307043930 18156677

